# Endogenous Selenoprotein P, a Liver-Derived Secretory Protein, Mediates Myocardial Ischemia/Reperfusion Injury in Mice

**DOI:** 10.3390/ijms19030878

**Published:** 2018-03-16

**Authors:** Hiroshi Chadani, Soichiro Usui, Oto Inoue, Takashi Kusayama, Shin-ichiro Takashima, Takeshi Kato, Hisayoshi Murai, Hiroshi Furusho, Ayano Nomura, Hirofumi Misu, Toshinari Takamura, Shuichi Kaneko, Masayuki Takamura

**Affiliations:** 1 Department of System Biology, Kanazawa University Graduate School of Health Medicine, 13-1 Takara-machi, Kanazawa, Ishikawa 920-8641, Japan; hirochada0310@gmail.com (H.C.); oto0803inoue@gmail.com (O.I.); ojiichan519@gmail.com (T.K.); takashin1974@gmail.com (S.T.); takeshikato@me.com (T.K.); sakurasoma1209@yahoo.co.jp (H.M.); hfurusho@m-kanazawa.jp (H.F.); ayanocchi.stier@gmail.com (A.N.); kaneko_s@m-kanazawa.jp (S.K.); mtakamura@m-kanazawa.jp (M.T.); 2 Department of Endocrinology and Metabolism, Kanazawa University Graduate School of Health Medicine, 13-1 Takara-machi, Kanazawa, Ishikawa 920-8641, Japan; hmisu@m-kanazawa.jp (H.M.); ttakamura@m-kanazawa.jp (T.T.)

**Keywords:** ischemia/reperfusion (I/R), selenoprotein P (SeP), reperfusion injury salvage kinase (RISK) pathway, hepatokine

## Abstract

Selenoprotein P (SeP), a liver-derived secretory protein, functions as a selenium supply protein in the body. SeP has been reported to be associated with insulin resistance in humans through serial analysis of gene expression. Recently, SeP has been found to inhibit vascular endothelial growth factor-stimulated cell proliferation in human umbilical vein endothelial cells, and impair angiogenesis in a mouse hind limb model. In this study, the role of SeP in ischemia/reperfusion (I/R) injury has been investigated. SeP knockout (KO) and littermate wild-type (WT) mice were subjected to 30 min of myocardial ischemia followed by 24 h of reperfusion. The myocardial infarct area/area at risk (IA/AAR), evaluated using Evans blue (EB) and 2,3,5-triphenyltetrazolium chloride (TTC) staining, was significantly smaller in SeP KO mice than in WT mice. The number of terminal de-oxynucleotidyl transferase dUTP nick end labeling (TUNEL)-positive nuclei was significantly lower in SeP KO mice than in WT mice. In addition, caspase-3 activation was reduced in SeP KO mice compared to that in WT mice. Furthermore, phosphoinositide 3-kinase/Akt and Erk levels were examined for the reperfusion injury salvage kinase (RISK) pathway. Interestingly, SeP KO significantly increased the phosphorylation of IGF-1, Akt, and Erk compared to that in WT mice after I/R. Finally, I/R-induced myocardial IA/AAR was significantly increased in SeP KO mice overexpressing SeP in the liver compared to other SeP KO mice. These results, together, suggest that inhibition of SeP protects the heart from I/R injury through upregulation of the RISK pathway.

## 1. Introduction

Ischemic heart disease is the leading cause of death in developed countries. Acute coronary syndrome (ACS) is the event that causes most deaths or new cases of heart failure [1]. Early revascularization therapy for occluded coronary arteries is the most effective treatment to reduce infarct size, and consequently, decrease mortality. On the other hand, post-ischemia reestablishment of coronary blood flow sometimes induces secondary detrimental effects, such as ventricular arrhythmia, myocardial stunning, and additional cell death [2]. Reperfusion itself can cause additional myocyte damage and death, which is typically defined as myocardial ischemia/reperfusion (I/R) injury. According to Yellon et al., I/R injury may be responsible for up to 50% of the final myocardial damage during ACS [3]. However, no specific clinically effective method is currently available to reduce I/R injury; thus, extensive research is focused on the cardioprotective signaling pathways, such as the reperfusion injury salvage kinase (RISK) pathway, including Akt and extracellular signal-regulated kinase 1/2 (Erk1/2) [4].

The insulin resistance and hyperinsulinemia in patients with type 2 diabetes are strong predictors of ischemic heart disease. In addition to elevating the incidence of ACS, diabetes is also associated with increased cardiac morbidity and mortality following the recanalization of an occluded coronary artery compared to the non-diabetics, partly due to an increased size of myocardial infarction [5]. A brief episode of ischemia is known to be associated with myocardial resistance to lethal I/R injury, but these cardioprotective effects against I/R injury are abolished in patients with diabetes [6,7]. Although the underlying mechanisms of this phenomenon are not fully understood, a growing body of evidence demonstrates that insulin resistance could influence the severity of I/R injuries.

Selenoprotein P (SeP; encoded by SELENOP in humans) is a selenium supply protein, primarily expressed in the liver [8,9]. SeP serves as a hepatokine that contributes to the onset of hyperglycemia in type 2 diabetes by imparting insulin signal transduction in the liver and skeletal muscle [10]. In contrast, SeP-neutralizing antibodies attenuate insulin resistance and improve hyperglycemia in mouse models of type 2 diabetes [11]. Recent evidence has shown that SeP impairs health-promoting effects of exercise by suppressing exercise-induced molecular adaptations in the skeletal muscle through the low-density lipoprotein receptor-related protein 1 [12]. A previous study also reported that SeP affects angiogenesis in mouse hind limb models [13], thereby suggesting that SeP exerts significant effect on ischemia. However, the relationship between SeP and myocardial I/R injury has not been previously investigated.

We hypothesized that the liver-derived secretory protein SeP contributes to I/R injury by acting on the RISK pathway. In this study, the role of SeP during myocardial I/R has been investigated using SeP knockout (KO) mice.

## 2. Results

### 2.1. SeP Gene Deletion Reduces I/R Injury in Mice

**Figure 1:**
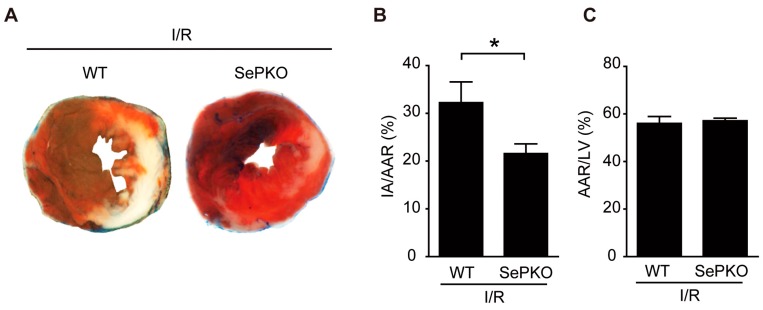
Selenoprotein P (SeP) knockout (KO) mice reduces I/R injury. SeP KO and control wild-type (WT) mice were subjected to 30 min of ischemia and 24 h of reperfusion. (**A**) Representative examples of myocardial infarction stained with Evans blue (EB) and triphenyl tetrazolium chloride (TTC) 24 h after reperfusion. EB-stained areas (blue) indicate non-ischemic regions; TTC-stained areas (red) indicate area at risk (AAR); EB/TTC-negative (white) areas indicate myocardial infarct area (IA). (**B**) The myocardial IA/AAR (percentage) is shown. (**C**) AAR/left ventricle (LV) size (percentage) is shown. Data represent means from at least five mice each. * *p* < 0.05.

To evaluate the role of SeP in mediating I/R injury, we applied I/R to SeP KO and WT mice. The mean area at risk (AAR), evaluated using 2,3,5-triphenyltetrazolium chloride (TTC) and Evans blue (EB) staining, in the left ventricle area, was similar in both SeP KO and WT mice (Figure 1C). *SeP* gene deletion led to a significant decrease in the myocardial infarct area/area at risk (IA/AAR) after I/R in SeP KO compared to that in WT mice (Figure 1A,B).

### 2.2. SeP Gene Deletion Reduces I/R-Induced Apoptosis in the Heart

**Figure 2:**
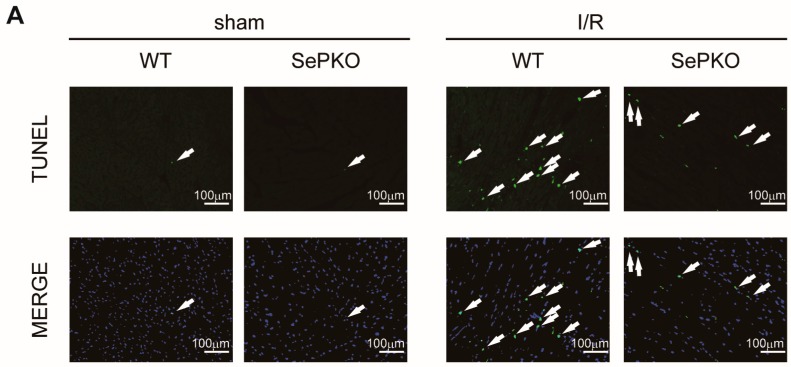

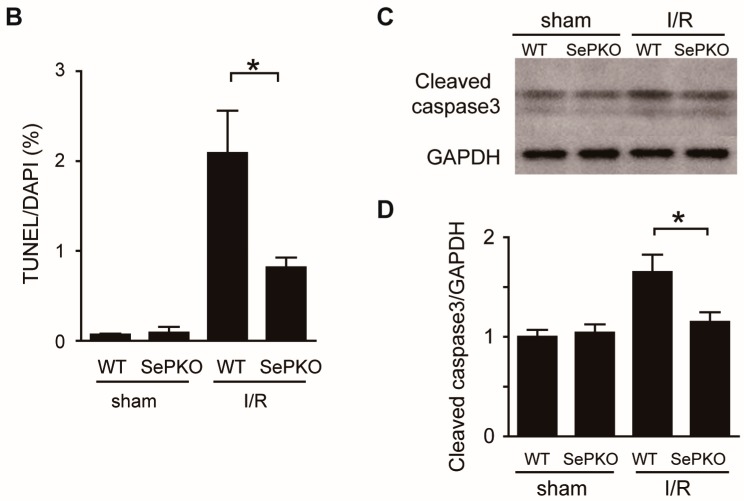
SeP KO mice reduces apoptotic cells after myocardial I/R. LV tissue sections were subjected to TUNEL and 4′,6-diamidino-2-phenylindole (DAPI) staining. (**A**) Representative examples of TUNEL-positive myocytes in the ischemic area. Arrows indicate TUNEL-positive myocytes. (**B**) The number of TUNEL-positive myocytes was expressed as a percentage of total nuclei detected using DAPI staining. Heart homogenates were prepared from SeP KO and WT mice subjected to 30 min of ischemia and 24 h of reperfusion. Immunoblot analyses were performed using (**C**) anti-cleaved caspase-3 and GAPDH antibody. (**D**) Results of quantitative analysis of cleaved caspase-3 are shown. Data represent means from at least five mice each. * *p* < 0.05.

I/R injury is associated with increased apoptosis in cardiac myocytes. To determine the extent of apoptosis, dUTP nick end labeling (TUNEL) staining was performed on the myocardium of mice subjected to 30 min of ischemia and 24 h of reperfusion. I/R significantly increased the number of TUNEL-positive cells in the treated mice at 24 h post-reperfusion, compared to that in sham-operated mice. Interestingly, the number of TUNEL-positive cells in SeP KO mice was significantly lower than that in wild-type (WT) mice (Figure 2A,B). Consistent with TUNEL staining, cleaved caspase-3 decreased in SeP KO mice compared to that in WT mice (Figure 2C,D). These results suggest that downregulation of SeP reduces I/R injury, accompanied by a decrease of apoptosis in the myocardium.

### 2.3. SeP Gene Deletion Increases the Phosphorylation of IGF-1 Receptor, Akt, Erk, and S6K

**Figure 3:**
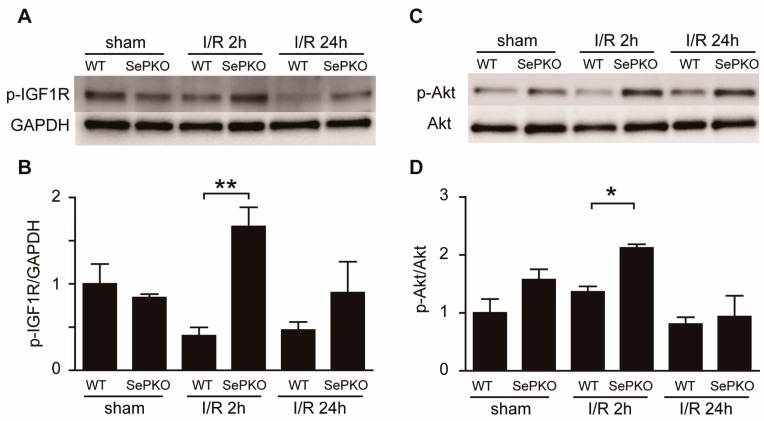

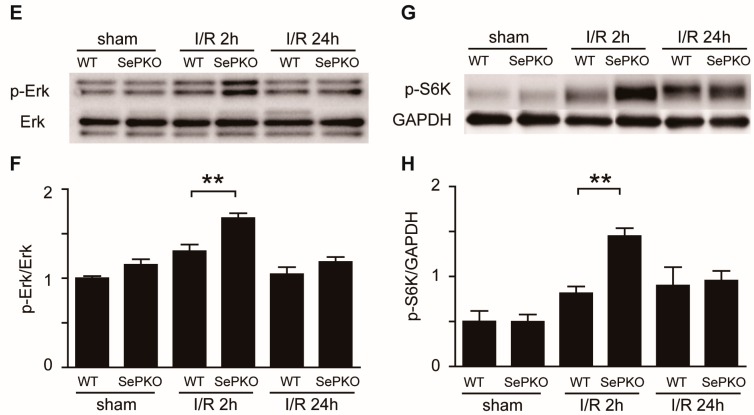
*SeP* gene deletion regulates the reperfusion injury salvage kinase. WT and SeP KO mice were subjected to 30 min of myocardial ischemia and 2 and 24 h of reperfusion. Representative Western blots of (**A**) phosphorylated-IGF1R and GAPDH, (**C**) phosphorylated-serine473-Akt and total Akt, (**E**) phosphorylated-Erk1/2 and total Erk1/2, and (**G**) phosphorylated-p70S6K and GAPDH. Quantification is shown in (**B**) p-IGF1R/GAPDH, (**D**) p-Akt/t-Akt, (**F**) p-Erk/t-Erk, and (**H**) p-S6K/GAPDH. Data are shown as means ± SEM from at least five mice each. * *p* < 0.05, ** *p* < 0.01.

Previous studies have shown that pro-survival signaling in ischemic hearts is mediated by the RISK pathway, involving the activation/phosphorylation of Akt and Erk. The phosphorylation levels of survival kinases from RISK (Akt, Erk, IGF, and S6K) were evaluated, and found to be similar in sham-operated SeP KO and WT mice. Interestingly, the phosphorylation levels of all four kinases were significantly upregulated in SeP KO mice compared to that in WT mice at 2 h of reperfusion (Figure 3).

### 2.4. SeP Induces Myocardial I/R Injury in Mice

**Figure 4:**
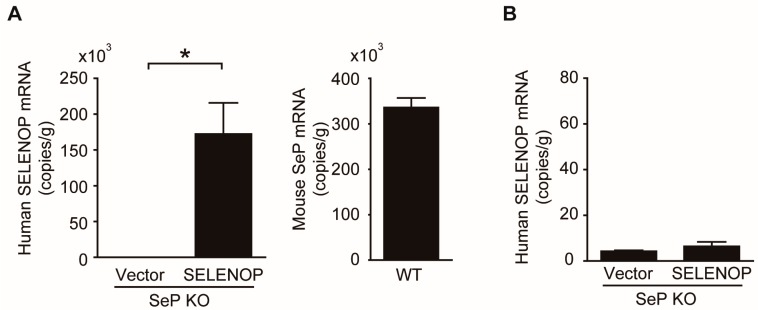

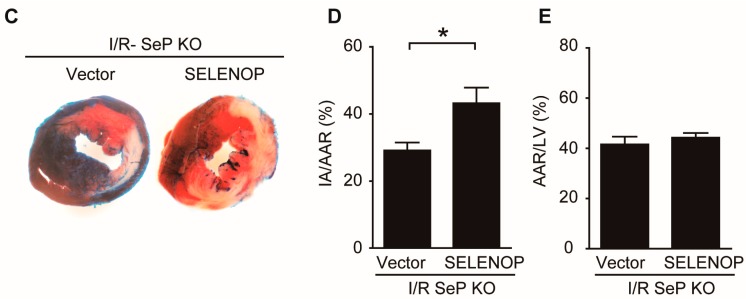
Hepatic overexpression of SeP enhances I/R injury. (**A**) Level of human SELENOP mRNA in the liver of SeP KO mice injected with plasmid DNA and that of mouse SeP mRNA in the liver of WT mice. (**B**) Level of human SELENOP mRNA in the heart of mice injected with plasmid DNA. SeP KO mice, injected with SELENOP plasmid and control into the tail vein, were subjected to 30 min of ischemia and 24 h of reperfusion. (**C**) Representative examples of myocardial infarction stained with EB and TTC after 24 h reperfusion (*n* = 3/control plasmid, *n* = 6/SELENOP plasmid). (**D**) The myocardial IA/AAR (percentage of IA/AAR) is shown. (**E**) AAR/LV size ratio is shown. Data represent means from at least five mice each. * *p* < 0.05.

To determine whether hepatic overexpression of SeP affects myocardial I/R, a hydrodynamic injection method was used to generate mice that overexpress human SELENOP mRNA in the liver of SeP KO mice. While the expression level of *SELENOP* gene was significantly elevated in the liver of these mice (Figure 4A), the levels were not significantly different in the heart (Figure 4B). Copy number of SELENOP in the liver of SeP KO mice was about half of that seen in the liver of WT mice (172.3 × 103 copies/g and 335.8 × 103 copies/g, respectively). Hepatic overexpression of SELENOP in SeP KO mice led to a significant increase in the myocardial IA/AAR, after I/R, compared to that in other SeP KO mice (Figure 4C–E).

### 2.5. SeP Impaired the RISK Pathway during I/R Injury in Mice

**Figure 5:**
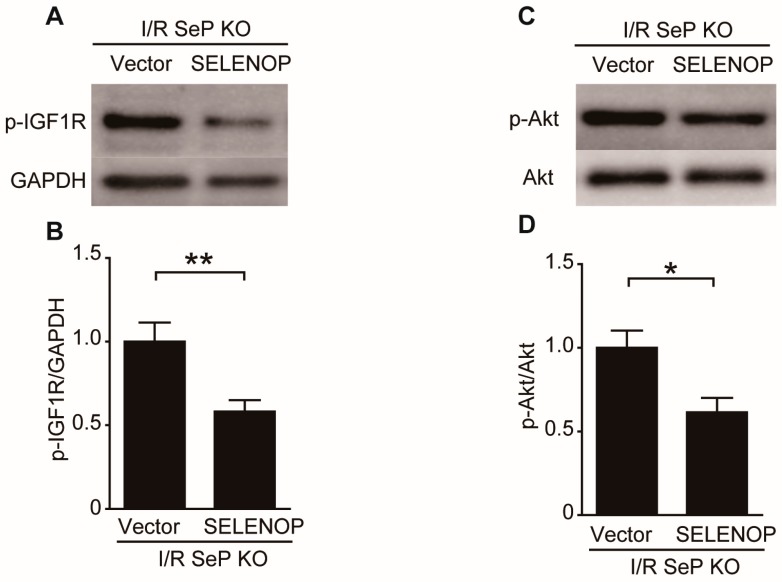

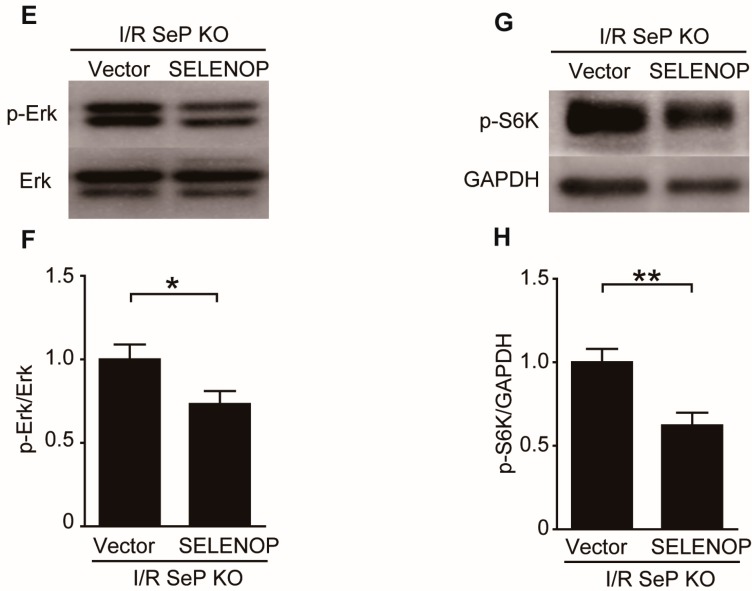
Hepatic overexpression of SeP impairs the reperfusion injury salvage kinase. Human SELENOP or control plasmid-administered SeP KO mice were subjected to 30 min of myocardial ischemia and 2 h of reperfusion. Representative Western blots of (**A**) phosphorylated-IGF1R and GAPDH, (**C**) phosphorylated-serine473-Akt and total Akt, (**E**) phosphorylated-Erk1/2 and total Erk1/2, and (**G**) phosphorylated-p70S6K and GAPDH. Quantification is show in (**B**) p-IGF1R/GAPDH, (**D**) p-Akt/t-Akt, (**F**) p-Erk/t-Erk, and (**H**) p-S6K/GAPDH. Data are represented as means ± SEM from at least five mice each. * *p* < 0.05, ** *p* < 0.01.

SeP deletion-induced phosphorylation of IGF1R, Akt, Erk, and S6K in the heart at 2 h after reperfusion was significantly less in the SeP KO mice overexpressing SeP, compared to the control vector-administered SeP KO mice (Figure 5).

## 3. Discussion

In the present study, we provided direct in vivo evidence of liver-derived secretory protein SeP mediating the deleterious effect of myocardial I/R injury. The main findings of this study are as follows: (1) SeP KO reduced infarct size after MI; (2) apoptosis after I/R in SeP KO mice was significantly reduced than that in WT mice; (3) gene deletion of SeP enhanced the phosphorylation of IGF-I, Akt, Erk1/2, and S6 kinase in the heart after myocardial I/R; (4) hepatic overexpression of SeP in SeP KO mice led to a significant increase in the infarct size following I/R compared to that in other SeP KO mice; and (5) hepatic overexpression of SeP reduced the activation of reperfusion injury salvage kinase (RISK) pathway in the SeP KO heart during myocardial I/R. These findings led us to conclude that *SeP* gene deletion attenuated myocardial I/R injury, possibly through its anti-apoptotic effect via activation of the RISK signaling pathway.

SeP is a liver-derived secretory protein that functions as a selenium transport protein. Hepatic gene expression levels of SeP have been reported to positively correlate with the severity of insulin resistance. SeP causes insulin resistance and hyperglycemia in diabetes, and treatment with purified SeP protein impairs insulin signal transduction [10]. Physiological concentrations of SeP inhibit VEGF-stimulated cell proliferation, tubule formation, and migration in human umbilical endothelial cells, leading to impaired angiogenesis [13]. However, no data demonstrating the role of SeP in the heart under pathophysiological conditions, such as myocardial I/R, is available. In this study, we found that deletion of *SeP* gene significantly reduced the myocardial I/R injury, hence implying a possible role of SeP in the injury. SeP is mainly produced by the liver, and found in high abundance in the serum [14]. Its expression level in the heart is less than that in the liver [15]. In this study, SeP KO mice, injected with SELENOP plasmid, were shown to have increased I/R-induced infarct size compared to those injected with control plasmid. These results suggest that the liver-derived SeP regulates I/R injury in the heart.

The mechanisms underlying myocardial I/R injury are complex. Although re-establishment of blood flow is essential, reperfusion may cause additional damage to the heart through a multitude of mechanisms [16]. One of the major cardioprotective target against myocardial I/R injury is the RISK pathway activation, consisting of phosphatidylinositol 3-kinase (PI3K)/Akt and Erk1/2 [17]. Our results demonstrated that deletion of *SeP* gene increased the phosphorylation of IGF-1/Akt and Erk/S6, while hepatic overexpression of SeP reduced SeP deletion-induced activation of the RISK pathway. These results suggest that the increase in I/R-induced infarct size by SeP was brought about by the inhibition of RISK pathway during I/R injury. Patients with diabetes are known to have impaired RISK pathway during myocardial I/R [18,19]. The expression levels of SeP in the liver are positively correlated with the severity of insulin resistance in diabetes, and SeP is known to reduce insulin-induced Akt phosphorylation in both liver and skeletal muscle in mice [10]. Thus, SeP possibly mediates the impairment of the RISK pathway during myocardial I/R in patients with diabetes. This hypothesis, however, requires further studies in a diabetic state following myocardial I/R injury.

The present study showed that deletion of *SeP* gene increases phosphorylation of IGF-1 in the heart following I/R, and reduces the I/R-induced infarct size. Previous studies revealed that IGF-1 plays a crucial role in protecting the heart from permanent coronary artery occlusion or I/R injury [20]. Intracoronary administration of IGF-1 reduces myocardial I/R-induced cardiomyocyte apoptosis in pigs [21]. Intramyocardial administration of IGF-1 also decreases the infarct size and cardiac dysfunction following myocardial infarction in rats [22]. IGF-1 signaling was impaired in the myocardium of a diabetic animal model [23]. From these findings, we speculated that SeP promotes I/R-induced infarction through the impairment of IGF-1 signaling in the heart.

Apoptosis is a highly regulated process of programmed cell death and contributes to myocyte cell death following I/R injury [24]. Classic apoptotic pathways are characterized by the activation of caspases, especially the executive caspase-3, and eventually cell death. Blocking the apoptosis process could prevent myocyte loss and reduce I/R-induced cardiac injury, thereby preventing the progression of heart failure [25]. In this study, we found that deletion of *SeP* gene decreased the number of TUNEL-positive cells and cleaved caspase-3 following I/R injury, compared to WT mice. The phosphorylated IGF-1 and Akt were increased in SeP KO mice following I/R, but decreased in WT mice. IGF-1/Akt signaling pathway is a typical cardioprotective pathway, activated by exercise, pressure overload, nutrients, and other stimulations [26]. Once activated, Akt may exert its anti-apoptotic effect through the phosphorylation of two categories of downstream substrates: the anti-apoptotic substrates, such as Bcl-2, and the pro-apoptotic substrates, such as Bax and caspase-3 [27]. Our results showed that the inhibition of cardiac apoptosis during I/R could be, at least partially, mediated by the activation of Akt signaling pathway. To determine the effect of SeP on cardiac myocyte apoptosis accurately, we need to perform further in vitro studies using hypoxia/re-oxygenation model.

In conclusion, we have clearly demonstrated that deletion of SeP attenuated the myocardial I/R injury. *SeP* gene deletion reduced infarct size, and decreased cardiac apoptosis. These cardioprotective effects in murine model of myocardial I/R are mediated by the activation of RISK pathway, resulting in a final reduction of myocardial apoptosis. Our findings suggest that SeP may be a promising target for the treatment of myocardial I/R injury.

## 4. Materials and Methods

### 4.1. Ethics Statement

All animal protocols were performed in compliance with the Guide for the Care and Use of Laboratory Animals in Kanazawa University, which strictly conforms to the Guide for the Care and Use of Laboratory Animals, published by the US National Institutes of Health (NIH, Bethesda, Rockville, MD, USA). The protocol was approved by the ethics committee of Kanazawa University (Approval NO AP-143146, 30 April 2014).

### 4.2. Animal Models and Experimental Procedures

SeP KO mice were produced by homologous recombination with genomic DNA cloned from a Sv129 P1 library and backcrossed to C57BL/6 strain, as previously described [28]. Heterozygous mice were used to generate SeP KO along with litter mate wild-type (WT) mice. Twelve-week-old male SeP KO and littermate WT mice were subjected to I/R or sham operation, as described. Briefly, a lateral thoracotomy was performed in anesthetized and ventilated mice, and a 7-0 suture was looped under the left descending coronary artery for induction of coronary artery occlusion for 30 min, followed by 24 h of reperfusion. The hearts were removed, and the area at risk (AAR) and infarct area (IA) were determined using EB and TTC staining [29].

### 4.3. Evaluation of Apoptosis

DNA fragmentation was detected in situ using terminal de-oxynucleotidyl transferase dUTP nick-end labeling (TUNEL), as described previously [30]. De-paraffinized sections were incubated with proteinase K, and DNA fragments were labeled with fluorescein-conjugated dUTP using TdT enzyme (Roche Molecular Biochemicals, Mannheim, Germany). The nuclear density was determined by manual counting of 4′-6-diamidino-2-phenylindole (DAPI)-stained nuclei in 10 fields for each animal. The number of TUNEL-positive nuclei was photographed by a fluorescent microscope (BZ-9000, Keyence, Tokyo, Japan) and counted by examination of the entire section, using a 40× objective.

### 4.4. Western Blotting

At 2 h and 24 h after reperfusion, the hearts of mice were harvested. A tissue from the anterior wall of LV was homogenized in radioimmunoprecipitation assay (RIPA) buffer containing 20 mmol/L Tris (pH 7.5), 10 mmol/L imidazole, 300 mmol/L sucrose, 25 mmol/L sodium fluoride, 10 mmol/L β-glycerophosphate, 100 mmol/L NaCl, 1 mmol/L Na_3_VO_4_, 1 mmol/L EGTA, 1 mmol/L EDTA, 2 µg/mL leupeptin, 1 µg/mL aprotinin, 1 mmol/L AEBSF, and 0.5 mmol/L DTT. The homogenates were centrifuged at 15,000× *g* for 5 min. Equal amounts of proteins were loaded, separated by electrophoresis on sodium dodecyl sulfate-polyacrylamide (SDS-PAGE) gels, and transferred onto a polyvinylidene fluoride membrane [31]. The following antibodies were used for further analysis: anti-cleaved caspase-3 (#19677, Sigma-Aldrich, Tokyo, Japan), anti-phospho-IGF-I receptor β (#3024s, Cell Signaling, Tokyo, Japan), anti-Akt (#9272, Cell Signaling), anti-phospho-Akt (#4060, Cell Signaling), anti-p44/42 MAPK (Erk1/2) (#9102, Cell Signaling), anti-Phospho-p44/42 MAPK (Erk1/2) (#9101, Cell Signaling), anti-phospho-p70 S6 kinase (Thr421/Ser424) (#9204, Cell Signaling), and anti-GAPDH (Santa Cruz Biotechnology, Santa Cruz, CA, USA).

### 4.5. Preparation of Human SELENOP Plasmids and Overexpression of SeP in Mice

The human SELENOP expression plasmids were provided by Kaketsuken (The Chemo-Sero Therapeutic Research Institute, Tokyo, Japan). The human *SELENOP* gene was cloned into the pBR322 expression vector and a control plasmid DNA was also produced. To generate SELENOP overexpression in mice, the plasmid was injected into the tail vein of SeP KO mice, as described earlier [13,32]. Briefly, 55 μg of human SELENOP pDNA or control pDNA in 1.8 mL of phosphate-buffered saline (pH 7.4, CaCl_2_-, MgCl_2_-) was injected within 10 s into the tail vein of SeP KO mice using a 26 G needle. Two days later, human SELENOP mRNA expression was measured in the liver and heart of the mice. SeP KO mice, injected with plasmid for two days, were next subjected to 30 min of ischemia and 24 h of reperfusion.

### 4.6. Total RNA Isolation and Quantitative Real-Time Polymerase Chain Reaction (QRT-PCR)

Total RNA was prepared from the hearts of mice using the RNeasy Fibrous Tissue Mini Kit (QIAGEN, Chatsworth, CA, USA) and from the livers using the QuickGene RNA Tissue Kit SII (FUJIFILM, Kanagawa, Japan) [33]. RT-PCR was carried out using TaqMan Gene Expression Assays and the Universal PCR Master Mix (Applied Biosystems, Foster City, CA, USA), which uses an ABI prism 7900HT Sequence Detection System (Applied Biosystems). The following TaqMan probes (Applied Biosystems) were used: human SELENOP (Hs01032845_m1) and mouse SeP (Mm00486048_m1).

### 4.7. Statistical Analysis

All values are expressed as mean ± standard error of the mean (SEM). Statistical analyses were performed using either Student’s *t*-test or one-way analysis of variance (ANOVA) followed by a post hoc Bonferroni–Dunn’s comparison test. A value of *p* < 0.05 was considered significant. Statistical analyses were conducted using GraphPad Prism (GraphPad Software, La Jolla, CA, USA).

## Abbreviations

SeP	selenoprotein P
I/R	ischemia/reperfusion
KO	knockout
WT	wild-type
IA/AAR	myocardial infarction/area at risk
EB	Evans blue
TTC	2,3,5-triphenyltetrazolium chloride
TUNEL	terminal deoxynucleotidyl transferase dUTP nick-end labeling
RISK	reperfusion injury salvage kinase
ACS	acute coronary syndrome
Erk1/2	extracellular signal-regulated kinase 1/2
